# Morphometric analysis of the mandibular incisive canal using cone beam computed tomography images

**DOI:** 10.1186/s13005-026-00594-1

**Published:** 2026-02-06

**Authors:** Yaser Safi, Zeynab Azizi, Sepideh Rahimian, Tahereh Dehghanian, Bita Heydarzadeh

**Affiliations:** 1 Department of Oral and Maxillofacial Radiology, School of Dentistry, Shahid Beheshti University of Medical Sciences, Tehran, Iran; 2 Department of Oral and Maxillofacial Radiology, School of Dentistry, Shahed University of Medical Sciences, Tehran, Iran

**Keywords:** Cone beam computed tomography, Dental implants, Mandibular incisive canal

## Abstract

**Background:**

The mandibular incisive canal (MIC), an anterior extension of the inferior alveolar canal, contains neurovascular bundles and is clinically significant. On cone beam computed tomography (CBCT) scans, it appears as a radiolucency with a radiopaque rim. This study aimed to investigate anatomical variations of the mandibular incisive canal using CBCT images from patients referred to treatment centers in Tehran during 2023–2024.

**Materials and methods:**

In this cross-sectional study, 345 CBCT scans (690 images) from patients referred to medical centers in Tehran were analyzed. The MIC’s length, diameter, and distances from anatomical landmarks were measured. Data were analyzed using descriptive statistics, chi-square tests, t-tests, ANOVA, and Mann-Whitney tests.

**Results:**

The mandibular incisive canal was identified in 74.5% of patients and 60.6% of CBCT images. The mean length and diameter of the MIC were 13.7 ± 3.90 mm and 1.65 ± 0.55 mm, respectively. No statistically significant differences in MIC visibility were found across age groups or between edentulous and dentate patients. Mean distances from the MIC’s superior border to the apices of premolars, canines, and incisors were 6.46 mm, 7.10 mm, and 8.99 mm, respectively. Mean distances from the MIC’s buccal cortical border to the buccal alveolar bone plate, from its inferior border to the mandibular inferior border, and from its lingual cortical border to the lingual alveolar bone plate were 2.60 mm, 8.72 mm, and 4.53 mm, respectively. A branch canal was observed in 79.5% of cases, with the MIC showing a downward direction in 64.8% and terminating at the canine apex in 58% of cases.

**Conclusion:**

Based on this study’s findings, due to anatomical differences in the mandibular incisive canal, careful evaluation of CBCT images is recommended before surgical procedures in the mandibular interforaminal region, to prevent damage to neurovascular bundles.

## Introduction

The mandibular incisive canal (MIC) is defined as the anterior continuation of the inferior alveolar canal beyond the mental foramen, housing neurovascular bundles in the anterior part of the mandible and supplies innervation to the mandibular incisor, canine, and first premolar teeth [1, 2]. Generally, the incisive branch of the mandibular canal runs obliquely downward and is divided into narrow canals at different levels [3]. These canals contain the terminal branches of nerves and vessels that nourish the teeth, the intraosseous space, and the gingival tissues. In cone beam computed tomography (CBCT) images, the MIC appears as a radiolucent region within trabecular bone, encircled by an opaque rim [4].

The use of CBCT was first reported by Mozzo et al. [5]. In recent decades, it has been recommended as an imaging modality for the maxillofacial region. CBCT differs from multi-detector computed tomography (MDCT), which is commonly used in the medical field. CBCT utilizes a cone-shaped X-ray beam that allows image acquisition in a single scan [6]. CBCT was introduced in dentistry by Arai et al. in 1997 and quickly gained acceptance among dentists. This imaging technique offers advantages such as easy image acquisition, high accuracy, reduced artifacts, lower radiation dose, cost-effectiveness, and faster imaging acquisition compared to MDCT. One disadvantage of CBCT, however, is its lower contrast resolution. The American Academy of Oral and Maxillofacial Radiology (AAOMR) recommends CBCT for periodontal treatments, maxillofacial surgery, and orthodontics [7].

The region between the mental foramina is generally considered safe for procedures such as implant insertion, bone graft removal from the chin, and genioplasty in orthognathic surgery [8, 9]. Several complications, such as edema, hematoma, and sensory disorders, have been reported during or after surgery in this region [10, 11]. Various researchers have investigated the extent of canal visibility and length, its distance from the tooth apex, the distance from the lower and upper borders of the mandible, and the buccal and lingual plates [12–14].

Few studies have investigated the anatomical and morphometric characteristics of the mandibular incisive canal in the Iranian population [15, 16]. Given the variable anatomical characteristics of the MIC, understanding its path, diameter, and position within the mandible is essential during anterior mandibular surgeries, such as implant placement, to prevent damage to neurovascular bundles. Studies have proven that the measurements obtained through CBCT images are accurate and reliable [17]. This study evaluates the MIC using a larger sample size, a wider age range, and CBCT images. Thus, the current study was conducted to investigate the morphometric analysis of the mandibular incisive canal using CBCT images concerning the diameter and position of the MIC in relation to various anatomic landmarks.

## Materials and methods

This was a retrospective cross-sectional study. The study protocol was approved by the institutional review board of Shahid Beheshti University of Medical Sciences (code no: IR.SBMU.DRC.REC.1398.002), and it was conducted in accordance with the Declaration of Helsinki and its subsequent revisions. The study was conducted in accordance with the STROBE statement.

This study evaluated CBCT scans that were collected from three oral and maxillofacial radiology centers in Tehran with exposure factors based on the patient’s size. Two systems were used: Scanora 3D (Sordex, Helsinki, Finland) with the isotropic voxel size of 0.25 mm and NewTom VGI (AFP, Verona, Italy) with the isotropic voxel size of 0.3 mm. Voxel size of 0.3 mm provides adequate accuracy for evaluating anatomical structures such as mandibular incisive canal in maxillofacial region while maintaining a lower radiation dose [18]. The field of view (FOV) was set according to the patients’ dental problems, which included implant insertion, orthognathic treatment, evaluation of impacted teeth, trauma, etc.

Estimating the sample size based on the standard deviation of the mandibular incisive canal length, as reported in the study by Pires et al. [17], which was 3.8, provided us with the largest sample, calculated as follows:


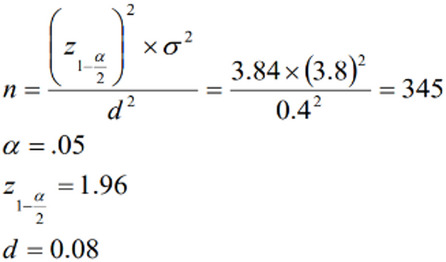


After meticulous checking, 345 images of appropriate quality were ultimately selected, in which the anterior mandible was bilaterally visible from the lower border to the crest, with no pathological lesions that would affect the position of the canal. The images were then reviewed by an experienced oral and maxillofacial radiologist.

The samples were classified based on age, sex (male or female), dental status (dentulous or edentulous), and the area under study (right or left side). The age range was 30–80 years, and the participants were divided into four subgroups: 30–49, 50–59, 60–69, and 70–80. Patients with at least one tooth in the anterior mental foramen were considered dentulous, while patients with no teeth in this region were classified as edentulous. All reconstructions and measurements were performed using the software associated with the same system. After processing, the interforaminal area was assessed in multi-planar and cross-sectional images.

**Fig. 1:**
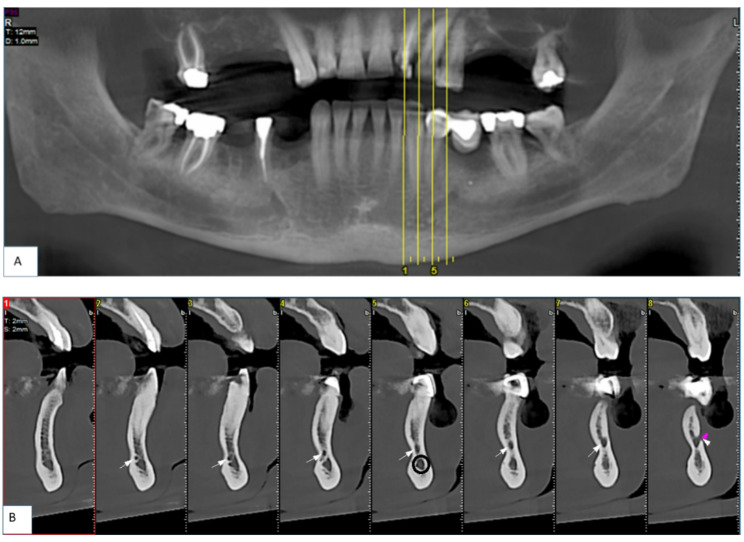
The MIC length measurement. (**A**) Reformatted panoramic with hashlines. (**B**) The mental foramen and interconnected vertical cross-sections showing the mandibular incisive canal. In this patient, the MIC extends from slice number 2 to slice number 7. Each cross-section was 2 mm, resulting in a 12 mm long incisive canal. Arrowhead: Mental foramen, Arrow: MIC, Black Circle: Trabecular bone

The canal visibility was evaluated; if visible, it was determined to be located on either the right or left side. The canal’s length was measured by counting the number of interconnected vertical sections after the anterior margin of the mental foramen or anterior loop to the last cross-sectional view visualizing the cortical opaque border of the canal (2-mm thick section) (Fig. 1) [16, 19]. Although reducing slice thickness increases the accuracy of canal length measurements, based on routine implant assessments, where 2 mm thick sections are typically evaluated, the present study utilized 2 mm slice thickness cross-sectional images to ensure clinical and practical applicability.

**Fig. 2:**
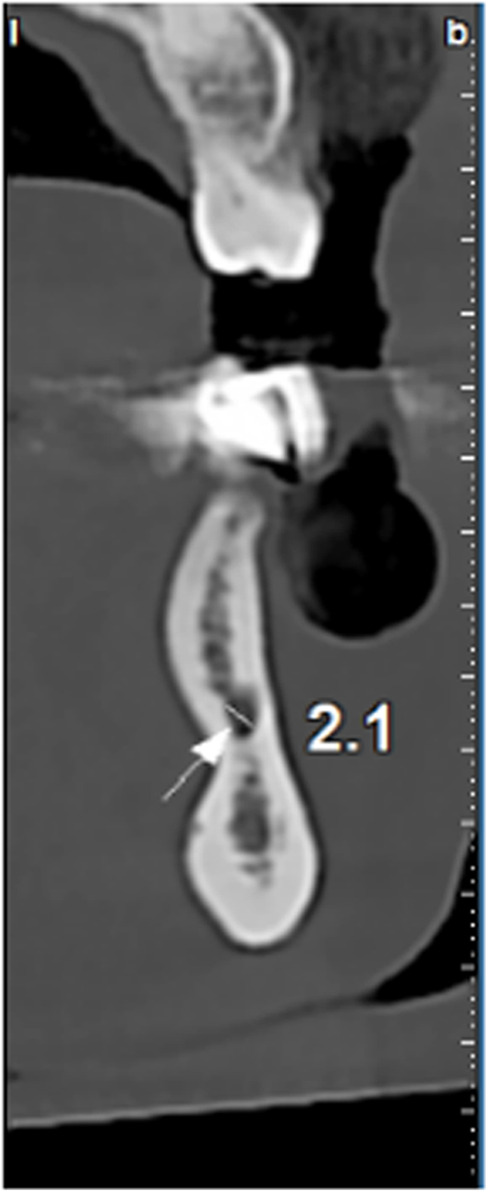
The cross-section that shows MIC (arrow) with a diameter of 2.1 mm

In order to obtain the canal diameter, the maximum distance between the internal cortical borders of the canal was measured on the sections of the image (Fig. 2) [20].

**Fig. 3:**
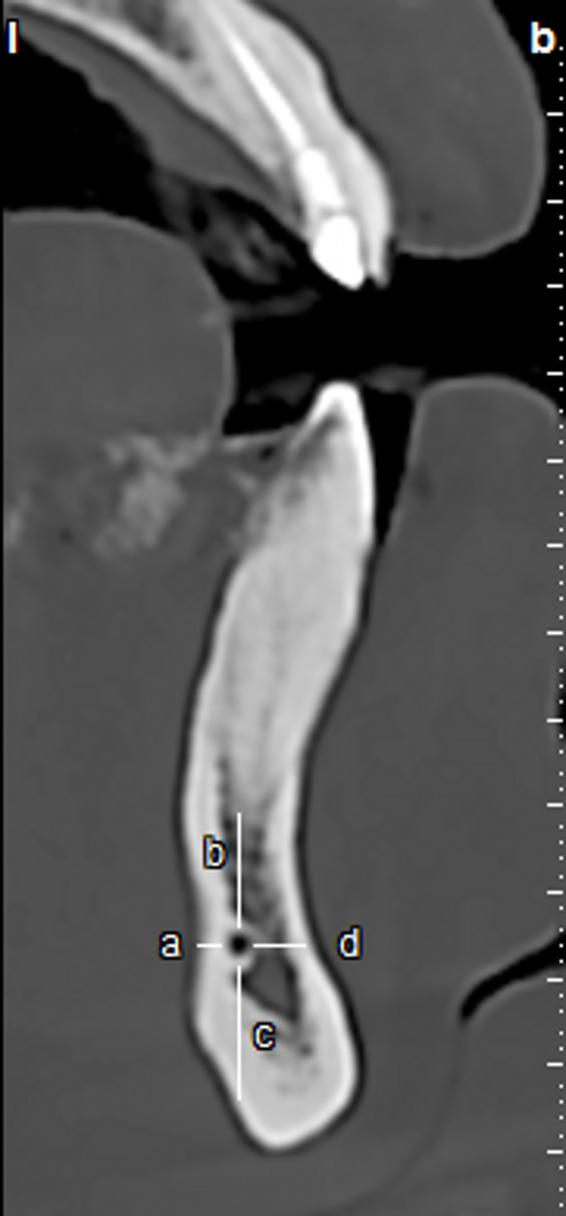
Measuring the spatial relationship and dimensions of the mandibular incisive canal. (a: The distance between the lingual cortical border of the canal and the lingual plate of the alveolar bone, b: The distance between the superior border of the incisive canal and the apices of the incisor, canine, and premolar teeth, c: The distance between the inferior border of the canal and the inferior border of the mandible, d: The distance between the canal and the buccal plate of the alveolar bone.)

The canal path was checked for being upward, straight, or downward [20]. The mean number of branches and the proximity of canal termination to the tooth apex were evaluated. In the area under study (right or left side), the distance between the superior border of the incisive canal and the apex of the incisor, canine, and premolar teeth was measured. In addition, the distance between the inferior border of the canal and the inferior border of the mandible, the distance between the buccal cortical border of the canal and the buccal plate of the alveolar bone, and the distance between the lingual cortical border of the canal and the lingual plate of the alveolar bone were measured (Fig. 3) [20].

The measurements were performed on the cross-sectional image, on which the canal diameter was calculated. The mean values of length, diameter, distance of the canal from the borders and tooth apices, as well as the number of branches, were calculated. The data were statistically analyzed using SPSS software, version 19. Considering the type of dependent and independent variables, Chi-square, t-test, and ANOVA were used as appropriate. An alpha value of 0.05 was chosen to test for any statistical significance. The normality of the data was evaluated with the Shapiro-Wilk test. If the data were abnormal, the non-parametric equivalent of the tests was employed. In the presence of any confounding factors, a multiple linear regression model was used. Due to ethical considerations, this study was conducted on archival images while preserving patients’ personal information. Intraclass correlation coefficients (ICC) were calculated on 20 samples to assess the intraoperator reliability.

## Results

### Demographic characteristics

The demographic characteristics of the study population are summarized in Table 1. The study included 345 patients (199 males and 146 females). Based on dental status, 260 patients were classified as dentate and 85 as edentulous.

**Table 1:** Demographic characteristics of the study population

Demographic characteristic	Number of patients	percentage
sex		
female	146	42.3%
male	199	57.7%
Age group (years)		
30–49	102	29.6%
50–59	125	36.2%
60–69	90	26.1%
70–80	28	8.1%
Dental status		
dentate	260	75.4%
edentulous	85	24.6%

### Reliability analysis

Intraclass correlation coefficient analysis based on two repeated measurements of 20 samples demonstrated good to excellent intra-observer reliability, with ICC values ranging from 0.73 to 0.99 for all parameters.

### Visibility of the mandibular incisive canal

Out of 345 patients and 690 reviewed images, 257 patients (74.5%) and 418 images (60.6%) showed the presence of the incisive branch, while 88 patients (25.5%) and 272 images (39.4%) did not. Based on the results of the Chi-square test, canal visibility was significantly related to sex (*p* = 0.024), with a higher prevalence in males (265 branches, 66.6%) (Table 2). McNemar’s test revealed that the frequency distribution of MIC visibility on the left and right sides was different (*p* = 0.041), with a higher prevalence on the left side (Table 3). Meanwhile, no significant relation was detected between canal visibility and age or dental status (dentate or edentulous) (*p* = 0.24 and *p* = 0.14, respectively) (Tables 4 and 5). Although no statistically significant association was found between canal visibility and age, the highest and lowest frequencies of visible incisive branches were observed in patients aged 50–59 years and those over 70 years, respectively.

**Table 2:** The frequency distribution of the visibility of the mandibular incisive Canal by sex

			Canal visibility		total
			visible	invisible	
sex	female	number	153	139	292
		percentage	52.40%	47.60%	100%
	male	number	265	133	398
		percentage	66.60%	33.40%	100%
total		number	418	272	690
		percentage	60.60%	39.40%	100%
*P*-value			0.024		

**Table 3:** The frequency distribution of the visibility of the mandibular incisive canal by area under study

			Canal visibility		total
			visible	invisible	
area under study	right	number	198	147	345
		percentage	57.40%	42.60%	100%
	left	number	220	125	345
		percentage	63.80%	36.20%	100%
total		number	418	272	690
		percentage	60.60%	39.40%	100%
*P*-value			0.041		

**Table 4:** The frequency distribution of the visibility of the mandibular incisive canal by age

			Canal visibility		total
			visible	invisible	
age	30–49	number	129	75	204
		percentage	63.20	36.80%	100%
	50–59	number	153	97	250
		percentage	61.20%	38.80%	100%
	60–69	number	106	74	180
		percentage	58.90%	41.10%	100%
	70–80	number	30	26	56
		percentage	53.60%	46.40%	100%
total		number	418	272	690
		percentage	60.60%	39.40%	100%
*P*-value			0.24		

**Table 5:** The frequency distribution of the visibility of the mandibular incisive canal by dental status

			Canal visibility		total
			visible	invisible	
dental status	dentulous	number	324	197	521
		percentage	62.20%	37.80%	100%
	edentulous	number	94	75	169
		percentage	55.60%	44.40%	100%
total		number	418	272	690
		percentage	60.60%	39.40%	100%
*P*-value			0.14		

### Morphometric characteristics of the mandibular incisive canal

**Fig. 4:**
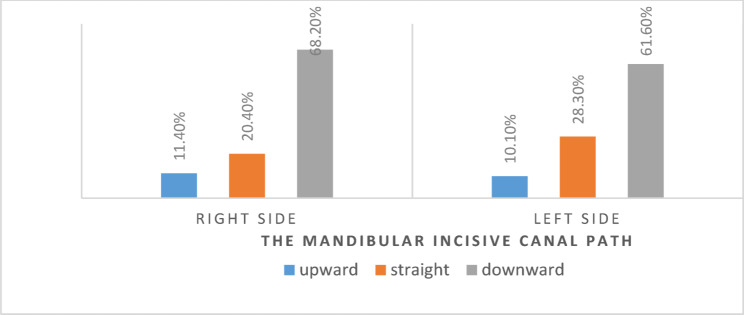
The percentage frequency distribution of the mandibular incisive canal path on the right and left sides

Out of 418 images with a visible canal, 272 images (64.8%) showed the canal running downward, 101 images (24.5%) showed it running straight, and 45 images (10.7%) showed it running upward. McNemar’s test revealed a significant difference in the percentage frequency distribution of the MIC path on the right and left sides (*p* = 0.044) (Fig. 4).

The Chi-square test showed no significant relation between the pathway and sex (*p* = 0.32). However, there was a significant difference between the canal pathway and age (*p* = 0.02), with the highest prevalence observed in patients aged 60–69, where 79 cases (73.8%) exhibited a downward path of the canal.

Regarding the terminal point, in 418 samples, 58% of incisive canals (*n* = 243) ended at the apex of the canine, 36.3% (*n* = 152) at the lateral apex, 4.5% (*n* = 19) at the central apex, and only 1.2% (*n* = 5) at the midline. Out of the 418 incisive canals, 334 cases had 1 branch, 72 cases had 2 branches, and 12 cases had 3 branches. Statistical tests did not reveal a significant difference between the various groups in terms of age, sex, dental status, and the involved side.

Table 6 displays the mean length and diameter of the branch, the distance from the superior border of the incisive canal to the apex of the incisor, canine, and premolar teeth, the distance from the inferior border of the incisive canal to the inferior border of the mandible, the distance from the buccal cortical border of the incisive canal to the buccal plate of the alveolar bone, and the distance from the lingual cortical border of the incisive canal to the lingual plate of the alveolar bone.

The length of the MIC was measured in the studied population, ranging from a minimum of 4 mm to a maximum of 26 mm, with a mean of 13.7 ± 3.90 mm. The diameter of the MIC ranged from a minimum of 1 mm to a maximum of 6 mm, with a mean of 1.65 ± 0.55 mm. The distance between the MIC and the apex of the premolar teeth was less than that of the canine and incisor teeth, indicating that the canal typically diverges from the tooth apices as it progresses toward the midline. The distance between the MIC and the buccal alveolar bone is shorter than the distance to the lingual alveolar bone; so, the mandibular incisive canal is generally located closer to the buccal alveolar bone. The distance between the MIC and the buccal, lingual and inferior borders of the mandible increased, respectively (Table 6).

**Table 6:** The number, mean, standard deviation (SD), minimum, and maximum values of the studied variables in the sample CBCT images

	A	B	C	D	E	F	G	H
mean	13.70	1.65	8.99	7.10	6.46	8.72	2.60	4.53
number	418	418	110	273	129	418	418	418
Standard deviation	3.90	0.55	2.12	2.04	2.05	1.59	1.10	1.56
minimum	4	1	3.9	0.1	2	1	1	1
maximum	26	6	13.5	13	13	13	10.5	10

A: Length of mandibular incisive canal

B: Diameter of mandibular incisive canal

C: Distance between the superior border of the incisive canal and the apex of incisor teeth

D: Distance between the superior border of incisive canal and the apex of canine teeth

E: Distance between the superior border of incisive canal and the apex of premolar teeth

F: Distance between the inferior border of incisive canal and the inferior border of mandible

G: Distance between the buccal cortical border of incisive canal and the buccal plate of alveolar bone

H: Distance between the lingual cortical border of incisive canal and the lingual plate of alveolar bone

According to the results of an independent t-test, the mean distance from the superior border of the incisive canal to the apex of the premolar teeth was the only variable in which males and females showed a significant difference (*p* = 0.031). This distance was greater in men (6.80 mm) than in women (6.04 mm).

## Discussion

In recent years, several studies have focused on natural landmarks and their structural variations to enhance the accuracy of surgical treatments. The inferior mandibular canal, which serves as the passage for neurovascular bundles, is one of the most critical landmarks due to its strategic position within the mandible. Damage to this structure remains one of the most serious complications associated with surgical interventions in the mandible [21].

Olivier first identified the incisive branch of the mandibular canal as the anterior extension of the inferior alveolar canal beyond the mental foramen [22]. In the cross-sectional images reviewed in the present study, the incisive branch of the mandibular canal was identified as a round radiolucent area in the trabecular bone, surrounded by an opaque rim. Given the importance of accurately assessing the interforaminal area during anterior mandibular surgeries to prevent damage to the mandibular incisive canal (MIC) and avoid unwanted complications. This study used CBCT to examine MIC variations in an Iranian population, providing data to guide safer surgical planning.

In the present study the MIC was visible in 74.5% of patients (60.6% of 690 CBCT images), which is lower than some studies. For instance, Martins et al. reported 100% visibility near the mental foramen, decreasing to 76–77% at 12–15 mm, while Nikkerdar et al. and Panjnoosh et al. found 97.62% and 97.5% visibility, respectively [15, 16, 23]. Our results are more consistent with Borghesi et al., who observed a marked reduction in visibility toward the midline, and Mousa et al., who reported 45.8% prevalence in an Egyptian population [24, 25]. Such discrepancies may be attributed to differences in study populations, imaging protocols, voxel size, and criteria used to define canal visibility. Importantly, these variations cannot be solely explained by racial factors, as conflicting results have also been reported within similar populations.

Gender-related and side-related differences were observed in the present study, with significantly higher MIC visibility in males and on the left side. These findings differ from those of Sahman et al., who reported no side-related differences [19]. No significant association was found between MIC visibility and age or dental status, which aligns with the findings of Guzmán et al. [26]. These differences may be attributed to variations in the study population, inclusion and exclusion criteria, and methodologies.

The mean diameter was 1.65 ± 0.55 mm in our study, consistent with Parnia et al. (1.47 ± 0.50 mm) but smaller than Nikkerdar et al. (1.89–1.94 mm) and Sahman et al. (1.91–1.94 mm) [16, 19, 27]. In our study, men had significantly wider MIC (*p* < 0.05), supporting findings by Sahman et al. and Guzmán et al., likely due to larger mandibular dimensions in men [19, 26].

In our study, the mean MIC length was 13.70 ± 3.90 mm (range 4–26 mm), longer than values reported by Barbosa et al. (7.7 mm), Pires et al. (7 mm), and Apostolakis and Brown (8.9 mm), but similar to Le et al. (12.83 ± 5.13 mm) [14, 17, 20, 28]. Differences in length measurement methods likely explain these variations.

In the present study, the vertical distance between the superior border of the mandibular incisive canal and the dental apices varied according to tooth type, being greatest in the incisor region, followed by the canine, and smallest in the premolar region. This distribution reflects the anatomical course of the MIC, which originates near the mental foramen and gradually diverges from the tooth apices as it progresses anteriorly toward the midline. Similar spatial trends have been reported by Barbosa et al. and Apostolakis and Brown, who demonstrated a closer relationship between the MIC and premolar apices compared with more anterior teeth [20, 29]. Clinically, this increasing separation toward the midline suggests a relatively lower risk of direct neurovascular injury in the incisor region, whereas the premolar and canine regions require greater caution during implant placement and other surgical procedures, underscoring the need for individualized CBCT assessment [21, 29].

In our study, the mean distance between the inferior border of the mandibular incisive canal and the inferior border of the mandible was 8.72 ± 1.59 mm, indicating that the canal is generally located superior to the inferior cortical plate within cancellous bone. Comparable vertical positioning of the MIC has been reported by Apostolakis and Brown and by Mousa et al., despite notable interindividual variability [20, 24]. Clinically, this vertical separation provides a protective bony margin; however, reduced mandibular height or advanced bone resorption may decrease this margin and increase the risk of neurovascular injury during implant placement or osteotomy procedures [21, 24].

Regarding buccolingual positioning, the mandibular incisive canal in the present study was located closer to the buccal cortical plate than to the lingual cortical plate. This finding is consistent with observations by Mousa et al. and Orhan et al. using CBCT imaging [6, 24]. Clinically, this buccal predominance increases the likelihood of canal injury or buccal cortical perforation during anterior mandibular surgical procedures, particularly in cases with limited buccal bone thickness. Variations in buccolingual distances among studies may reflect differences in mandibular cross-sectional morphology and population-specific anatomical characteristics; nevertheless, the present findings highlight the importance of careful buccolingual assessment of the MIC during implant planning and other surgical interventions.

In the present study, the MIC most commonly terminated at the canine apex (58%), a finding that carries important clinical implications. Sahman et al. also reported variability in the anterior termination of the canal, underscoring that the interforaminal region cannot be uniformly considered safe [19]. From a dental perspective, frequent termination near the canine highlights the increased risk of nerve injury during implant placement, apical surgery, or bone harvesting in this region, reinforcing the necessity of precise preoperative imaging.

In our study, there was no significant difference between MIC visibility in dentate and edentulous patients (*p* = 0.14), highlighting its relevance for all surgical candidates. In dentate patients, the canal’s proximity to tooth apices increases the risk of nerve damage during implant placement, as noted by Taşdemir et al. [11]. In edentulous patients, bone resorption may elevate the canal closer to the alveolar crest, raising risks during grafting or osteotomies, as described by Sener et al. and Safi et al. [10, 30]. Preoperative CBCT is thus essential for both groups to map the MIC and avoid issues like hematoma or sensory loss, as reported by Barbosa et al. and Safi et al. [8, 31].

This study contributes valuable data on MIC anatomy in an Iranian population, which remains underrepresented in the literature. The relatively large sample size, use of two CBCT systems (0.25–0.3 mm voxel size) and comprehensive evaluation of canal morphology, course, and spatial relationships enhance the reliability of the findings. These insights distinguish this work from other studies [32, 33]. However, confusion between the MIC and other intraosseous canals, particularly in the lingual mandible [33], may lead to overestimation of canal length. Additionally, reduced visibility near the midline may be attributed to narrowing of the canal, limitations in image resolution, or partial volume effects [24].

The incisive branch of the mandibular canal contains the neurovascular bundles that innervate the anterior mandible. These bundles may extend into the bone marrow spaces of the mandible in the absence of a clear incisive canal or through narrow, fine canals. As a result, some researchers have overlooked the presence of a distinct incisive canal, considering the interforaminal area to be safe. However, several studies emphasize the importance of determining the location, distance, and diameter of the incisive branch of the mandibular canal before performing any surgery in this region. This is especially critical when the mandibular incisive canal has a particularly wide diameter, which can pose serious risks, particularly during implant surgeries. Direct trauma to the incisive neurovascular bundle can result in sensory disorders, edema, and hematoma [15].

Given the considerable anatomical variability of the MIC, strict adherence to established safety margins during chin bone harvesting and other anterior mandibular procedures is essential. Nevertheless, the present findings suggest that reliance on generalized guidelines alone may be insufficient. Instead, individualized CBCT-based assessment should be considered mandatory to reduce the risk of MIC injury during anterior mandibular procedures.

## Limitations and recommendations

This study has limitations that should be acknowledged. First, all measurements were performed by a single observer, which may introduce observer-related bias. However, this approach also ensured consistency in measurement methodology, and the high intra-observer reliability demonstrated by the intraclass correlation coefficient analysis supports the reproducibility of the measurements. Second, the retrospective design of the study limited control over image acquisition parameters and patient selection. Third, although CBCT provides detailed three-dimensional visualization of the mandibular incisive canal, variations in canal morphology and course may still exist within the studied population, and less frequent anatomical patterns may not have been fully represented. In addition, the study was conducted in an Iranian population, which may limit the generalizability of the findings to other populations. Finally, the absence of clinical correlation precludes direct assessment of the clinical implications of the morphometric findings. Future prospective studies incorporating standardized imaging protocols, larger and more diverse populations, and clinical validation are recommended.

## Conclusion

Within the limitations of this study, cone beam computed tomography proved to be a reliable imaging modality for the assessment of the mandibular incisive canal. The MIC was visible in a considerable proportion of the studied population, with significant associations observed with sex and canal side, while no significant relationship was found with age or dental status. The morphometric findings demonstrated considerable variability in canal length, diameter, course, and terminal point, with the canal generally located closer to the buccal alveolar bone. These findings emphasize the importance of careful radiographic evaluation of the anterior mandible prior to surgical and implant-related procedures to minimize the risk of neurovascular injury.

## Data Availability

The datasets used and/or analyzed during the current study are available from the corresponding author on reasonable request.
